# TMEM16A alternative splicing coordination in breast cancer

**DOI:** 10.1186/1476-4598-12-75

**Published:** 2013-07-16

**Authors:** Ifeoma Ubby, Erica Bussani, Antonio Colonna, Giuseppe Stacul, Martina Locatelli, Paolo Scudieri, Luis Galietta, Franco Pagani

**Affiliations:** 1 Human Molecular Genetics, International Centre for Genetic Engineering and Biotechnology, Trieste, Italy; 2 Gorizia Hospital, Presidio Ospedaliero di Gorizia, Gorizia, Italy; 3 U.O.C. Genetica Medica, Istituto Giannina Gaslini, Genova, Italy; 4 Present address: NCCS, National Cancer Centre Singapore, Singapore, Singapore

**Keywords:** TMEM16A isoforms, Alternative splicing, Splicing coordination, Breast cancer

## Abstract

**Background:**

*TMEM16A*, also known as *Anoctamin*-*1*, is a calcium-activated chloride channel gene overexpressed in many tumors. The role of *TMEM16A* in cancer is not completely understood and no data are available regarding the potential tumorigenic properties of the multiple isoforms generated by alternative splicing (AS).

**Methods:**

We evaluated *TMEM16A* AS pattern, isoforms distribution and Splicing Coordination (SC), in normal tissues and breast cancers, through a semi-quantitative PCR-assay that amplifies transcripts across three AS exons, 6b, 13 and 15.

**Results:**

In breast cancer, we did not observe an association either to AS of individual exons or to specific *TMEM16A* isoforms, and induced expression of the most common isoforms present in tumors in the HEK293 Flp-In Tet-ON system had no effect on cellular proliferation and migration. The analysis of splicing coordination, a mechanism that regulates AS of distant exons, showed a preferential association of exon 6b and 15 in several normal tissues and tumors: isoforms that predominantly include exon 6b tend to exclude exon 15 and vice versa. Interestingly, we found an increase in SC in breast tumors compared to matched normal tissues.

**Conclusions:**

As the different *TMEM16A* isoforms do not affect proliferation or migration and do not associate with tumors, our results suggest that the resulting channel activities are not directly involved in cell growth and motility. Conversely, the observed increase in SC in breast tumors suggests that the maintenance of the regulatory mechanism that coordinates distant alternative spliced exons in multiple genes other than *TMEM16A* is necessary for cancer cell viability.

## Introduction

*TMEM16A* (also known as *ANO1*, *DOG1*, *ORAOV2* or *TAOS2*) is a member of the Anoctamin family of membrane proteins, which consists of ten components (known as *TMEM16A*-*K* or *ANO1*-*10*) that share a highly conserved structure with eight transmembrane domains and cytosolic amino- and carboxy-termini domains. In 2008, three independent groups identified *TMEM16A* as a bona fide Ca^2+^-activated Cl^-^ channel (CaCC) essential for a variety of physiological functions including neuronal and cardiac excitation, olfactory and sensory signal transduction, vascular tone and pain perception [1,2]. *TMEM16A* is expressed in many tissues that are known to express CaCCs: bronchiolar epithelial cells, pancreatic acinar cells, proximal kidney tubule epithelium, retina, dorsal root ganglion sensory neurons, and sub- mandibular gland [3-5]. In addition to epithelia, *TMEM16A* is robustly expressed in interstitial cells of Cajal, which are responsible for generating pacemaker activity in smooth muscle of the gut [6-8].

*TMEM16A* is a sensitive biomarker for the diagnosis of gastrointestinal stromal tumors (GISTs) and it is overexpressed in several cancers including urinary bladder cancer, esophageal cancer, prostate cancer, breast cancer, head and neck squamous cell carcinoma (HNSCC), parathyroid tumors and ovarian tumors [9-13]. However, its role in tumor cell proliferation and migration is not completely understood. In cellular models, *TMEM16A* has been reported to positively affect cell proliferation and/or migration [9,12-16]. Conversely, Wang et al. [17] showed a negative effect of cell proliferation by preventing cell cycle transition from the G0/G1 phase to the S phase via inhibition of cyclin D1 and cyclin E expression [17]. Several mechanisms have been reported that associate changes in Ca^2+^-dependent channel activities with its tumorigenic potential are unknown: prevention of cell cycle transition from the G0/G1 phase to the S phase via inhibition of cyclin D1 and cyclin E expression [17], activation of MAPK kinases with promotion of ERK1/2 and cyclin D1 activation [9] and activation of EGFR and CAMK signalling [13]. In general, similar to other channels such as Ca(2+)-activated K(+) channels and voltage-gated Cl(−) or K(+)channels, *TMEM16A* might affect cell motility and proliferation though dynamic changes in the cellular volume [18-21].

Alternative splicing of *TMEM16A* can generate multiple protein isoforms with different electrophysiological properties affecting the voltage and Ca^2+^-dependence of the channel [22]. The most important AS occurring on *TMEM16A* transcripts consist in skipping/ inclusion of three exons 6b, 13 and 15. Other minor splicing events affecting exon 1, 10, 14 and 18 have also been reported, leading to the formation of several TMEM16A transcripts [23,24]. We have previously reported the relative percentage of inclusion of each exon relative to the total amount of *TMEM16A* transcripts showing that in several normal human tissues, transcripts that predominantly include exon 6b, also tend to lack exon 15 in the same mature transcripts and vice-versa [22]. However, the relative proportion of each isoform that take into account the real association between the AS events on the same transcript is unknown.

In the *TMEM16A* gene and thus on the nascent transcripts, the three exons, 6b, 13 and 15 are located relatively far apart (exons 6b and 15 are located at 50 kb). Comparison of data from transcript sequencing efforts, EST/cDNA sequences and microarray profiling experiments have provided evidence for AS coordination between multiple exons within a single gene [25-28]. Indeed, distant alternative splicing events on the same gene can be correlated, through a mechanism known as intragenic Splicing Coordination (SC) [27,29,30]. The presence of multiple splice variants on the same transcript, as found in *TMEM16A*, can highly increase its potential to generate multiple transcripts. However, as AS events are generally analyzed separately through PCR amplification of short regions, this correlation is difficult to establish and have not been explored in tumors. The mechanism that regulates the coordinate selection of AS events in the same gene involves both *cis*-acting regulatory elements present on the nascent transcripts and Polymerase II -dependent transcriptional elongation [27].

In the present study, using a PCR-assay that amplifies transcripts across the three AS exons, we found that *TMEM16A* AS of exons 6b and 15 are coordinated in several normal tissues and that this coordination increases in breast tumors. AS of individual exons was not altered in breast tumor and overexpression of the *TMEM16A* isoforms in a controlled cellular system had no effect on proliferation and migration. These results suggest that *TMEM16A* expression and splicing does not confer a positive general effect on cell growth and motility. The unexpected increase of SC in breast tumors suggest that cancer cells maintain the regulatory mechanism that coordinates distant alternative spliced exons on the same transcript, a process that, by acting on multiple genes, may be important in tumor cell viability.

## Results

### Identification of *TMEM16A* mRNA isoforms in normal human tissues

**Figure 1:**
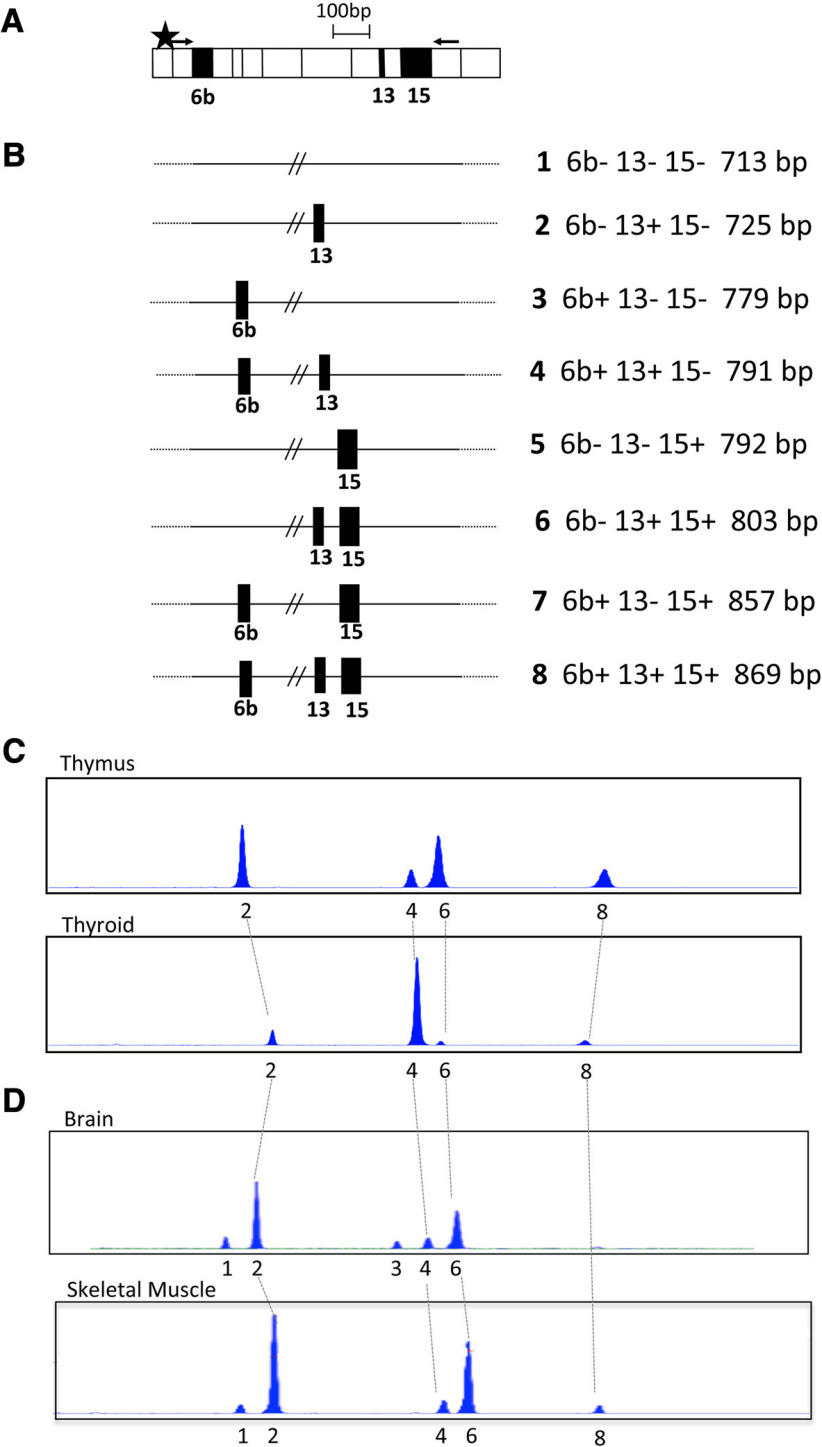
**Identification of *TMEM16A *isoforms in normal adult human tissues through capillary electrophoresis. A**, schematic representation of the *TMEM16A* mRNA showing the position of the AS exons (black boxes), and the oligonucleotide used in RT-PCR experiments. RNA was amplified with 803D^FAM^ and 1894R primers. **B**, cartoon indicating the eight possible *TMEM16A* transcript variants as a result of combination of exon 6b, 13 and 15 inclusion and/or exclusion. The expected sizes of RT-PCR products are indicated on the right. **C**, typical electrophoregram of the RT-PCR products, evaluated by capillary electrophoresis of tissues that express isoforms with exon 13 inclusion. Each peak corresponds to the isoforms indicated in B. **D**, typical electrophoregram of the RT-PCR products of tissues that express the isoforms without exon13. The identity of the bands was verified by directed sequencing.

To study the expression of the three *TMEM16A* AS exons, we previously used RT-PCR analysis, with primers that amplify each exon separately [22]. In normal human tissues, we observed significant changes in AS for exons 6b and 15, whereas exon 13 was minimally skipped only in two tissues (brain and skeletal muscle). In addition, several normal adult human tissues that predominantly include exon 6b tend to exclude exon 15 from the mature transcript [22]. To assess the relative abundance of the *TMEM16A* isoforms, we set up a denaturing capillary electrophoresis of fluorescent-5′ end labelled RT-PCR (Figure 1A). In this assay, the long-range PCR amplifies *TMEM16A* transcripts that include the three AS events. Due to the possible combinations of the three alternatively spliced exons 6b, 13 and 15, eight mRNA isoforms of different molecular size are expected to originate from the long range PCR (Figure 1B). The assignment of each peak to a specific splice variant was based upon comparison with standard molecular weight (MW) markers and direct sequencing of the bands eluted from the agarose gels. In the majority of tissues, we detected four splice variants of 725 bp, 791 bp, 803 bp and 869 bp, which correspond to 6b-13 + 15-, 6b + 13 + 15-, 6b-13 + 15+ and 6b + 13 + 15+ isoforms, respectively (isoforms 2, 4, 6 and 8 in Figure 1B and Additional file 1: Figure S1). Figure 1C illustrates a typical electropherogram of two tissues that contains these four isoforms. As expected and previously reported [22], we observed the appearance of additional bands of 779 bp and 713 bp (Figure 1D), which originate from exon 13 skipping and correspond to 6b + 13-15- and 6b-13-15- isoforms respectively (isoforms 1 and 3), in only two tissues, namely brain and skeletal muscle. The two isoforms 4 and 5, that correspond to 6b + 13+ 15- and 6b- 13- and 15+ could not be clearly distinguished by size (791 bp vs 792 bp) we assumed for quantitative purposes, that, in the samples where exon 13 is completely included, which represent the majority of tissues, they corresponds to band 4 only.

**Figure 2:**
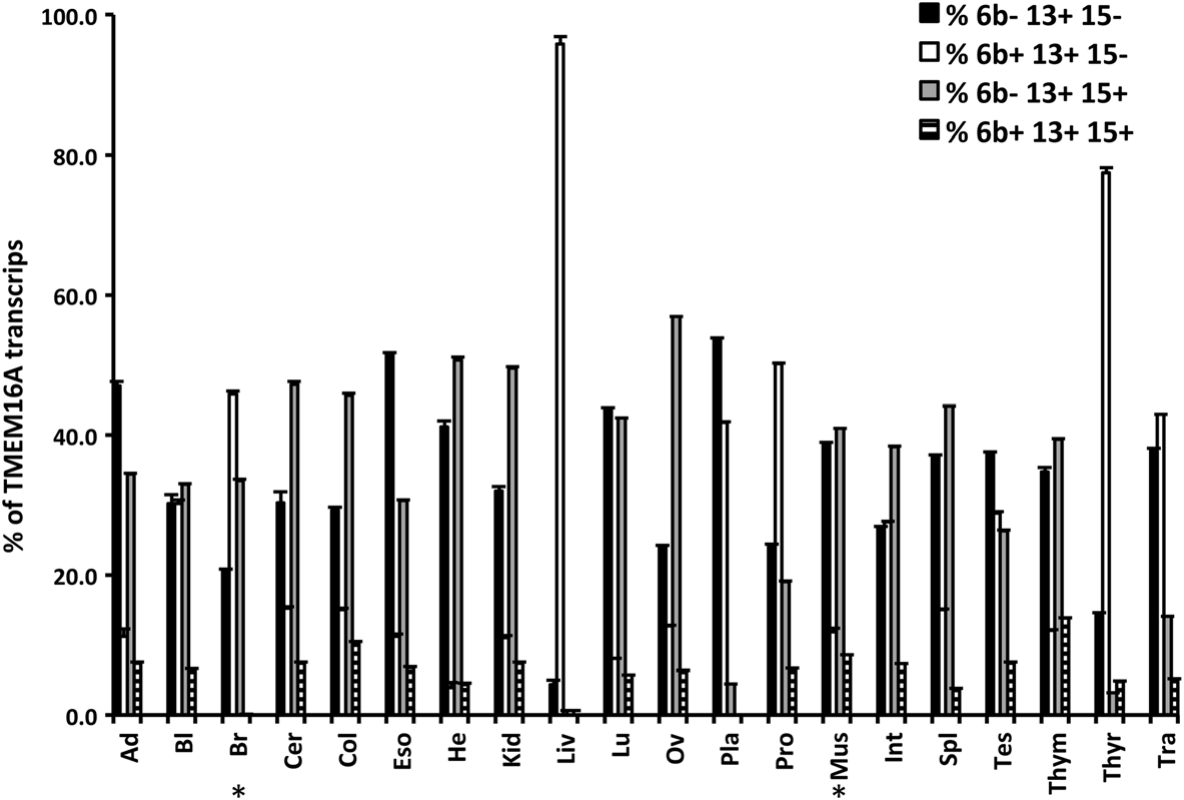
**Quantification of *TMEM16A *isoforms in normal adult human tissues.***TMEM16A* isoforms in 20 normal adult human tissues were separated using a capillary electrophoresis system as described in Figure 1 and percentages were determined from the total area of each peak. Data are expressed as mean ± SD of three independent experiments. *, brain and skeletal muscle showed additional minor 13- isoform, not reported in the graph. Ad = Adipose; Bl = Bladder; Br = Brain; Cer = Cervix; Col = Colon; Eso = Esophagus; He = Heart; Kid = Kidney; Liv = Liver; Lu = Lung; Ov = Ovary; Pla = Placenta; Pro = Prostate; Mus = Skeletal Muscle; Int = Small Intestine; Spl = Spleen; Tes = Testes; Thym = Thymus; Thyr = Thyroid; Tra = Trachea.

The percentage of each isoform was calculated as the area under the peaks, and Figure 2 shows the pattern of expression of the four major *TMEM16A* isoforms in 20 human normal adult tissues. The different peak height in the electrophoregram is a rough estimation of the expression levels of TMEM16A in the different tissues. The three most represented isoforms identified were 6b-13+ 15-, 6b + 13 + 15- and 6b-13+ 15+. Thus from this we can infer with approximation the tissue specific expression. The relative proportion of these isoforms varied significantly between tissues: for example, in lung 6b-13+ 15- and 6b-13+ 15+ were the most expressed whereas in liver and thyroid tissues, 6b + 13 + 15- was more prevalent, representing approximately 95% and 80%, of the total transcripts respectively (Figure 2). The isoform that contains all the three exons, 6b + 13+ 15+ was present in a lower amount in all samples. To verify that in our experimental conditions, the long range PCR does not preferentially results in amplification of selected isoforms, we compared the percentage of exon inclusion calculated with the long range PCR with the percentage obtained through amplification of individual exons (Additional file 2: Figure S2A). Results shown in Additional file 2: Figure S2B, exhibited a significant correlation between the percentages of exon 6b and 15 inclusions calculated with the two PCR amplification methods.

### *TMEM16A* alternative splicing of individual exons and of specific isoforms does not change in breast tumors

**Figure 3:**
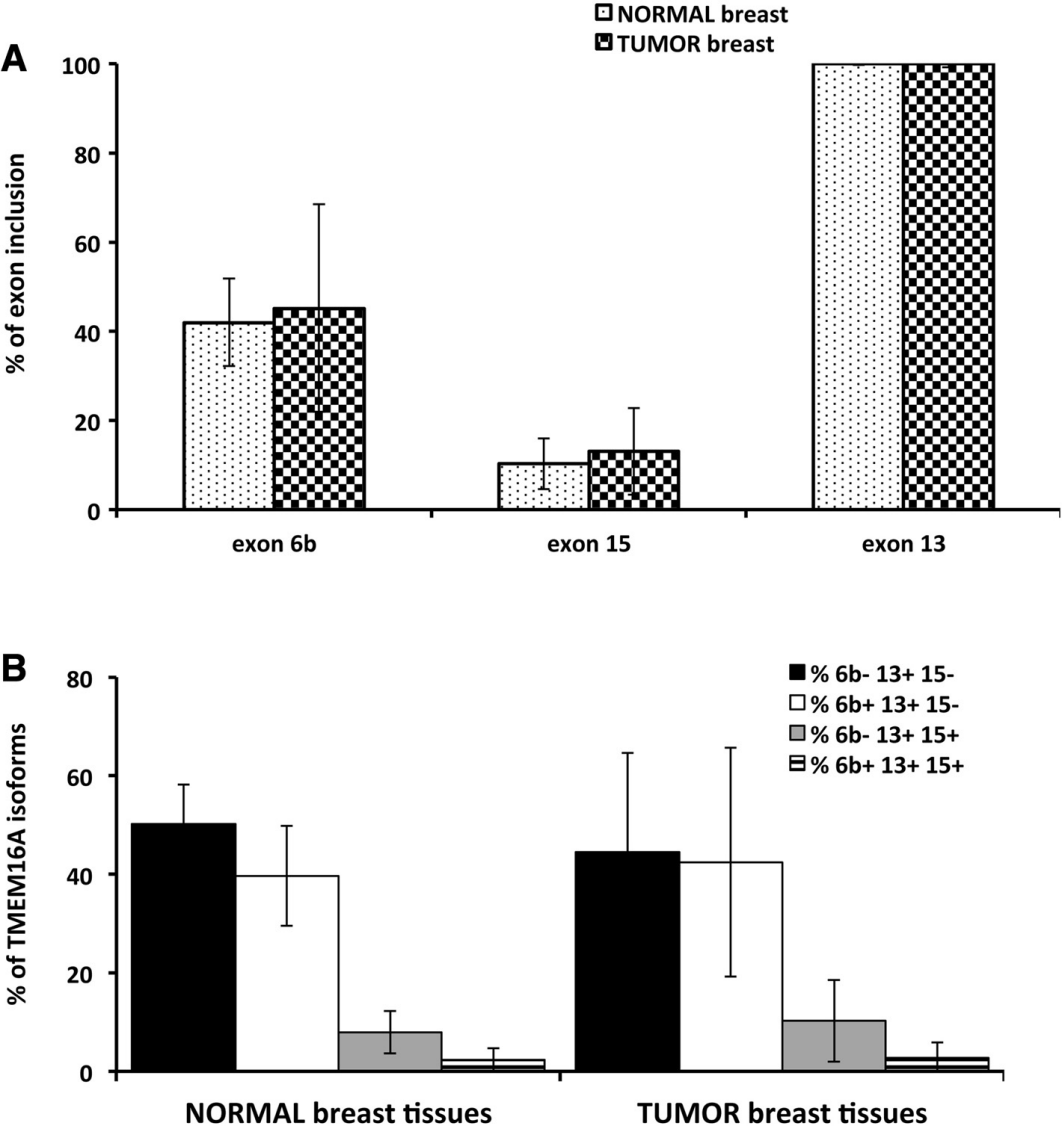
***TMEM16A *alternative splicing in human normal and cancer breast tissues. A**, individual analysis of the three alternatively spliced exons, 6b, 13 and 15, in normal and tumor breast tissues. The percentage is expressed as means ± SD, based on at least three independent capillary electrophoresis analyses. **B**, *TMEM16A* isoforms profiles in normal and tumor breast tissues. The percentage is expressed as means ± SD, based on at least three independent capillary electrophoresis analyses.

To explore the role of the different *TMEM16A* isoforms in tumors, we next analyzed 18 breast tumors and in their corresponding normal breast tissues obtained from the same surgical specimen. The histological and receptor status of the tumors shown in Additional file 3: Table S3 and the absence of tumor cells in the controls were verified by histological analysis. Expression of *TMEM16A* isoforms in the 36 paired tumor-normal breast tissues was evaluated by RT-PCR amplification of transcripts across the three AS events as done for the normal human tissues (Figure 1). All the samples showed four transcripts of 725 bp, 791 bp, 803 bp and 869 bp, which correspond to 6b-13 + 15-, 6b + 13 + 15-, 6b-13 + 15+ and 6b + 13 + 15+ isoforms respectively (isoforms 2, 4, 6 and 8 in Figure 1B). No band with skipping of exon 13 was detected. The data were analyzed by considering the percentage of inclusion of each exon on the total transcript (Figure 3A) and evaluating individually the different *TMEM16A* isoforms (Figure 3B). In the normal breast, exon 6b, 15 and 13 were present in approximately 40%, 10% and 100% of the *TMEM16A* transcripts respectively and these percentages did not change significantly in the tumors (Figure 3A). Similarly, the analysis of the distribution of the four mRNA isoforms showed no significant differences between the normal samples and the tumors (Figure 3B). Most of the transcripts in both cases corresponded to 6b- 13+ 15- and 6b + 13+ 15- isoforms (40-50%). Approximately 10% of transcripts correspond to 6b- 13+ 15+ isoform and 5% to the isoform that include all the exons (6b + 13 + 15+). The scatter of the standard deviation (SD) in tumor samples was increased in comparison to normal breast tissues, indicating a higher variability of the four isoforms distribution in cancer. All together these results indicate that neither the individual AS events nor the derived isoforms are associated to breast tumors.

### Overexpression of *TMEM16A* isoforms in HEK293 cells do not affect cell migration and cell proliferation

**Figure 4:**
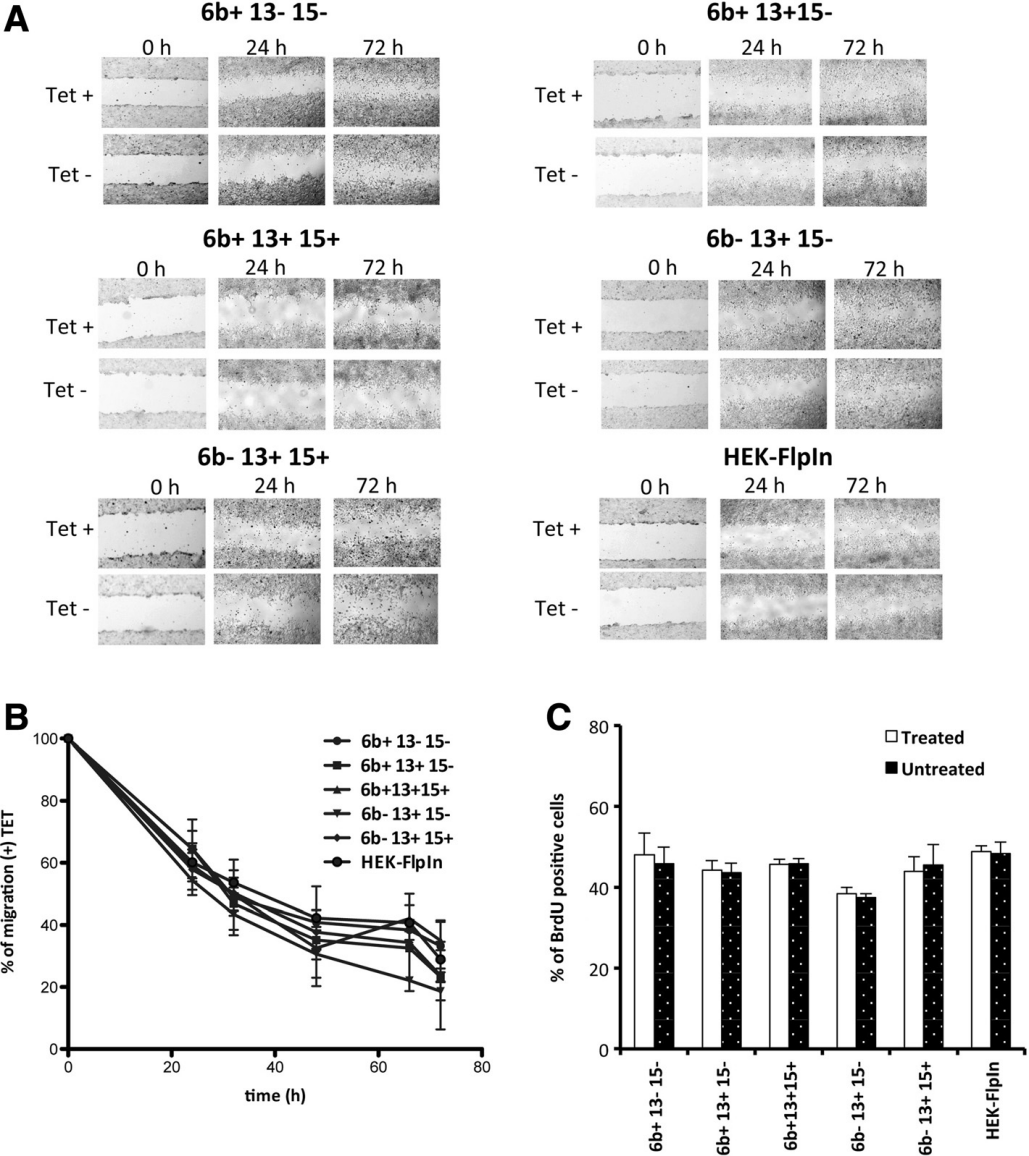
**Cell migration and proliferation assay of *TMEM16A *variants. A**, Representative images of wound healing in a scratch assay with inducible expression of *TMEM16A* variants in HEK293 cells cultured with (Tet+) or without (Tet−) tetracycline (0.1 μg/ml). Original magnification, 4x. (Scale bars: 5 μm). **B**, Quantification of the fraction of the wound that remains uncovered by the migratory cells as a function of time for cell treated with (Tet+) or without (Tet−). **C**, Cellular proliferation assay, BrdU staining of cells expressing *TMEM16A* variants (treated without or with tetracycline). Data represent the % of BrdU^+^ cells and are the mean ± SD of three independent experiments.

To determine the possible role of the different *TMEM16A* isoforms on migration and proliferation, we generated stable clones in HEK293 Flp-In cells. We selected the four most expressed isoforms detected in breast tumor samples (Figure 3B) and the additional isoform 6b + 13- 15- present in normal brain and skeletal muscle that lacks exon 13 (Figure 1). In the HEK293 Flp-In Tet-ON system, the presence of a unique copy of the transgene at the identical chromosomal site and driven by the inducible tetracycline (Tet) promoter, allow a precise and comparable evaluation of the biological properties of the different *TMEM16A* isoforms. In all recombinant clones, Tet addition induced expression of the corresponding *TMEM* isoform (Additional file 4: Figure S4). To assess *TMEM16A* channel activity, we used the halide-sensitive yellow fluorescent protein (HS-YFP) assay [3]. HEK293 cells transfected with the various *TMEM16A* isoforms responded to tetracycline treatment with a very large Ca^2+^-activated anion transport activity (Additional file 5: Figure S5). In contrast, control cells treated under the same conditions showed virtually no anion transport. To evaluate the effect of these TMEM16A isoforms on cell migration, we performed wound-healing assay. Confluent HEK293 cells monolayer (two for each isoform) were wounded with a sterile pipette tip (scratch assay) and wound healing (re-closure of the scratch) was followed by time lapse microscopy. Cells were incubated in the presence or absence of tetracycline for 72 h. Clones expressing the different *TMEM16A* isoforms (Figure 4A) did not show any significant difference in their capacity to migrate into the wounded area compared to the control HEK293 Flp-In empty cells (Figure 4A). Tet alone did not affect the percentage of migration between each clone and between the different clones (Figure 4B). We next evaluated whether overexpression of the *TMEM16A* isoforms could affect cell proliferation. For this purpose, we determined the incorporation of BrdU in untreated and Tet- treated HEK293 Flp-In Tet-on cells. We did not observe any significant difference in the percentage of BrdU positive cells among the different isoforms nor relative to the control cells (Figure 4C).

### Splicing Coordination of *TMEM16A* AS is higher in breast tumors

**Figure 5:**
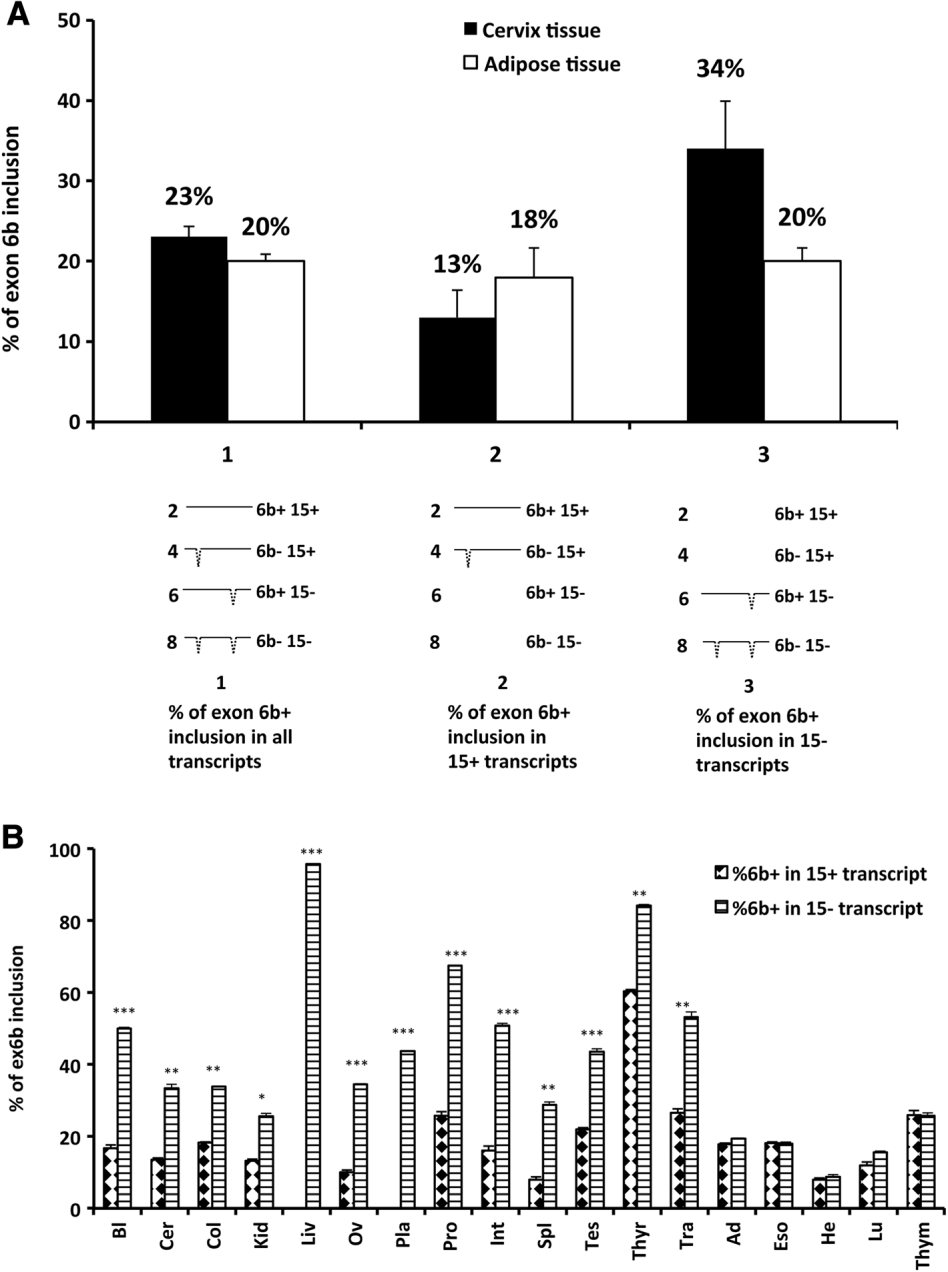
**Splicing coordination of *TMEM16A *mRNA isoforms in normal adult human tissues. A**, example of a positive (cervix) and a negative (adipose tissues) case of SC. The top of the panel shows the percentage of exon 6b inclusion in transcripts that include or exclude exon 15. Lane 1: percentage of exon 6b inclusion considering all the four isoforms. Lane 2: percentage of exon 6b inclusion in the isoforms that contain only exon 15. Lane 3: percentage of exon 6b inclusion in the isoforms that lacks exon 15. The identity of the isoforms considered in each calculation is depicted below each lane. **B**, Percentage of exon 6b inclusion in transcripts that contain or lack exon 15. Quantification of exon 6b inclusion associated to exon 15 inclusion or exclusion in 18 normal adult human tissues. Statistically analysis was performed using paired Student’s t-test (*p < 0.05; p < 0.01**; p < 0.001***). Ad = Adipose; Bl = Bladder; Cer = Cervix; Col = Colon; Eso = Esophagus; He = Heart; Kid = Kidney; Liv = Liver; Lu = Lung; Ov = Ovary; Pla = Placenta; Pro = Prostate; Int = Small Intestine; Spl = Spleen; Tes = Testes; Thym = Thymus; Thyr = Thyroid; Tra = Trachea.

To evaluate the relationship between the two major AS events on the same transcript and the presence of a possible intragenic Splicing Coordination (SC), we compared the percentage of exon 6b inclusion in transcripts that either included exon 15 or not. Significant differences in these percentages were considered as indicative of the presence of *TMEM16A* isoforms in which the two AS events are coordinated. The principle of the analysis along with a graphic representation of the distribution of the TMEM16A isoforms in two representative normal tissues, cervix and adipose (a positive and a negative case of coordination, respectively) is shown in Figure 5. In these two examples, the overall percentage of exon 6b inclusion is similar in cervix (23%) and in the adipose tissue (20%) (lane 1, in Figure 5A). However, the distribution of the exon 6b abundance in 15+ and 15- transcripts was different between them. In fact, considering only the isoforms that include exon 15 (lane 2), the corresponding level of inclusion of exon 6b was reduced to 13% in cervix but not in adipose tissue. Similarly, in transcripts without exon 15 (lane 3), the inclusion of exon 6b increased to 34% in cervix but did not change in adipose tissue. Thus, even if the total percentage of exon 6b was similar in the two tissues, only one (i.e. Cervix) showed some degree of splicing coordination. The analysis showed that exon 6b has a preferential association with transcripts in which exon 15 is skipped and vice versa. The comparison of exon 6b inclusion in transcripts that included exon 15 to transcript without it in 18 normal tissues showed that the two AS exons 6b and 15 were coordinated in 14 out of them. Coordination was evident in Bladder, Cervix, Colon, Kidney, Liver, Ovary, Placenta, Prostate, Small Intestine, Spleen, Testes, Thyroid and Trachea, although Adipose, Esophagus, Heart, Lung, and Thymus tissues were not coordinated (Figure 5B). The analysis considering the distribution of exon 15 inclusion associated to exon 6b isoforms gave comparable results (Additional file 6: Figure S6).

**Figure 6:**
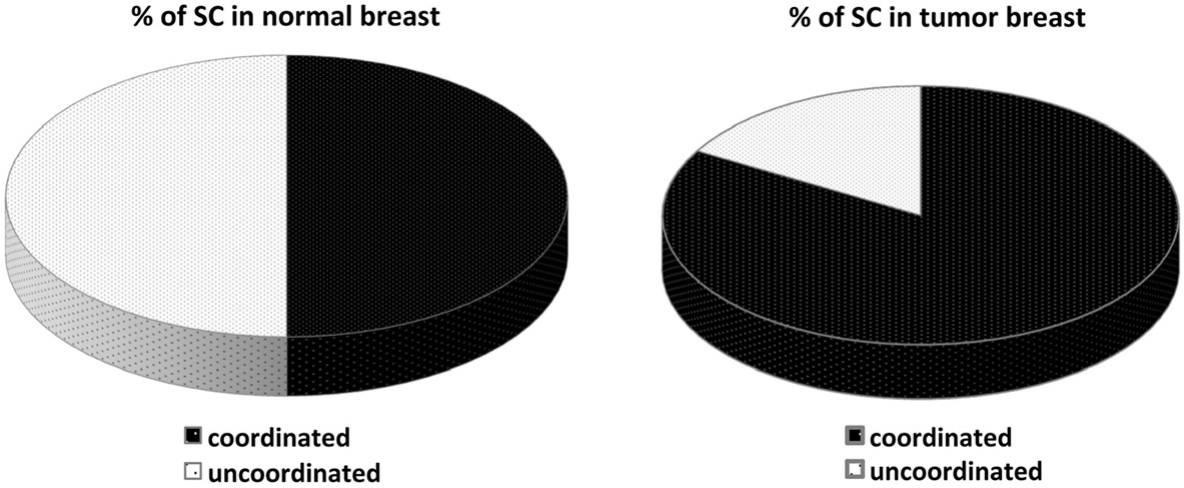
**Alternative splicing Coordination of *TMEM16A *in normal and breast cancer tissues.** Analysis of SC was performed in 18 normal breasts and corresponding 18 tumors.

Next, we evaluated the *TMEM16A* splicing coordination in mRNA derived from normal and tumor breasts samples. This analysis revealed that in 9 out of 18 normal breast tissues (50%), the two AS events were coordinated indicating the presence of an individual variability. Interestingly, the intragenic splicing coordination was present in 15 breast tumors (84%) (Figure 6) and this increase in splicing coordination is due to the fact that 6 normal breasts that were not coordinated turn to be coordinated in the corresponding cancer tissues (Additional file 7: Figure S7). Thus, in *TMEM16A* transcripts derived from normal breast, the exon 6b and 15 showed evidence of splicing coordination in 50% of the samples and this percentage increase to 84% in tumors.

## Discussion

In this paper, we have evaluated the potential role in breast cancer of the different *TMEM16A* isoforms generated through alternative splicing. The analysis of the alternative splicing pattern *in vivo* in breast tumors and the functional analysis of the isoforms overexpressed in a cellular model strongly suggest that the *TMEM16A* Ca^2+^-dependent Cl^-^ channel activities are not directly involved in tumorigenesis. More precisely, the lack of effect on HEK293 growth and motility indicates that, if *TMEM16A* has a role on cancer progression, it is not due to a general effect on the regulation of cell cycle and migration, as previously proposed [9,12-15]. However, this does not exclude that *TMEM16A* has a more cancer-specific role that is relevant to tumor growth *in vivo*. On the other hand, in *TMEM16A* transcripts derived from several normal tissues and breast tumors, inclusion/exclusion of the two distant alternatively spliced exon 6b and 15 is not independent but occur in a coordinated manner on a single transcript. As this splicing coordination is significantly increased in breast tumors, the regulatory mechanism that coordinates distant alternative spliced exons on the same transcript is maintained in these cells. We speculate that maintenance of the mechanisms that control SC is necessary for regulation of AS events in other genes directly involved in tumor cell viability.

Several studies have associated *TMEM16A* expression with cancer [9,31-33]. However, as tumors may originate from cells that normally express *TMEM16A*, like GIST, which is thought to derive from the interstitial cells of Cajal [33,34], it is not clear if this channel is just an associated marker or if it directly promotes tumorigenesis. In addition, the mechanism that associates its Ca^2+^-dependent Cl^-^ activity to cellular proliferation is unclear, and several studies reported contradictory effects on proliferation and/or migration. In basilar smooth muscle cells, where *TMEM16A* is abundantly expressed, its downregulation promotes proliferation and accordingly restoration of *TMEM16A* activity was suggested to be beneficial on hypertension-associated cardiovascular disease such as stroke [17]. On the contrary, other studies reported that *TMEM16A* promotes cell migration alone [35] or both proliferation and migration [9,12,14,15]. In xenografts, loss of *TMEM16A* through siRNA-mediated silencing was reported to inhibit tumor growth [9,13]. As a result of this positive effect on proliferation, and in contrast to the effect on smooth muscle cells [17], the therapeutic inhibition of *TMEM16A* activity was proposed for the treatment of cancers [9,13]. In this study, the analysis of the different *TMEM16A* isoforms generated through AS *in vivo* in breast cancer and in a controlled cellular model, does not support a direct role of the major *TMEM16A* isoforms in tumorigenesis. The evaluation of the different functional properties of isoforms derived from AS is not an easy task, in particular if referred to migration and proliferation. To avoid clonal variability that can affect transgene expression due to the numbers of copies integrated and site of integration, we used the inducible HEK293 Flp-In Tet-ON system and the results clearly show that the *TMEM16A* isoforms per se are not sufficient to promote or inhibit proliferation and migration (Figure 4). Even if our result does not support a direct role of *TMEM16A*, the different isoforms might indirectly affect proliferation and migration in a cell-type specific manner. Accordingly, to affect proliferation and/or migration, *TMEM16A* expression would require expression of additional factors, and this association could be at the basis of the contradictory effects reported in different cell types. This cell type specificity might be critical for the development of therapeutic strategies, as different pathological conditions and target cells may differently respond to inhibition or restoration of the *TMEM16A* activity, as previously reported [9,13,17]. The HEK293 Flp-In Tet-ON system we have developed here, expressing the different isoforms in a regulated manner, can represent an interesting model to identify, with a high-throughput screening method, those factors that not only promote or inhibit proliferation in a TMEM-dependent manner but also potential AS-specific networks.

An interesting and novel aspect of our study is the identification in several normal tissues of *TMEM16A* SC and the observation that this coordination increases in breast tumors (Figure 6). In common with ~ 25% of human genes, *TMEM16A* has more than one alternative splicing possibility and accordingly can generate several isoforms. Through a mechanism known as intragenic splicing coordination, [27,28,36] the association between alternative spliced events is not random, as we have found for exon 6b and 15 in *TMEM16A*. In normal breast, approximately 50% of samples showed SC, which increased to 84% in tumors. Normal mammary glands express *TMEM16A*[37-39] and the individual hormonal status might have an important effect on its AS pattern and SC thus explaining the variability observed in normal tissues. In several genes, hormones modulate AS [40,41] but the effect on splicing coordination is unknown. Through histological evaluation, we do not have evidence of the presence of tumor cells in normal samples, but normal glands showed a variable relative abundance of different cell types (like epithelial, stromal and adipocytes) and this might also contribute to the individual variability.

The fact that the expression of the two AS exons 6b and 15 is coordinated in the majority of *TMEM16A* transcripts derived from tumors (84%), suggest that cancer progression is not associated with a relaxation of this phenomenon. The mechanisms underlying splicing coordination are largely unknown. Fibronectin (FN) [27] and *slo*-*1* BK potassium channel [30] are well-studied examples of genes with intragenic AS coordination. In these cases, mutations that affect one AS exon had a profound effect on the other AS events, with both 5’ to 3’ polarity or bi-directionality for *FN* and *Slo*-*1*, respectively. In addition, proper coordination of intragenic alternative splicing has been found essential for normal physiology of the *slo*-*1* gene in vivo in *Caenorhabditis elegans*[30]. Based on this evidences a mechanism that lead to preferential expression of given alternate exon combination was engaged. Recruitment of splicing factors with direct interactions between the RNA-protein complexes from distinct splice sites, RNA secondary structure determinants and changes in Pol II elongation or processivity have been suggested to be involved [27,30]. Another interesting hypothesis to explain intragenic AS coordination might involve the formation of chromatin loops. Chromatin loops have been reported to physically link promoters to the end of the gene in order to facilitate Pol II-dependent transcription [42-45]. These loops can also occur in introns, as demonstrated for the 85-Kb long *BRCA1* gene in human cell lines and in mouse mammary tissues [31]. Interestingly, these loops change upon estrogen stimulation and during lactational development [31]. Thus it is possible that loops that put in contact distant alternatively spliced regions on the same gene might have a role in splicing coordination and possibly regulated by hormonal status. In this manner, Pol II engaged in transcription at different AS exons might physically communicate with reciprocal influence on the corresponding splicing decisions. This event might be still operative in tumor cells in order to preserve cancer viability. More deep molecular studies are required to unravel the basic mechanism of intragenic splicing coordination and understand their role in cancer.

What could be the advantage of a tumor cell to maintain an intragenic splicing coordination? As none of the *TMEM16A* isoforms directly affect proliferation or migration (Figure 4), it is possible that during tumorigenesis the cell would have to maintain active and efficient the mechanism involved in splicing coordination. This might not be specifically useful for the *TMEM16A* expression but for coordinating splicing of other genes directly involved in proliferation or apoptosis such as *CD44* gene, *Ron* gene or *FGFR2* gene [46-49].

In conclusion, this study has improved our understanding of TMEM16A splicing coordination with the identification and characterization of a non-random distribution of the mRNA isoforms in normal adult human breast tissues and tumor.

In this context, the maintenance of splicing coordination will be required for preventing a massive transcript alteration that will lead to cell death and thus the study of the basic mechanism involved might be useful for the identification of novel targets for therapeutic intervention.

## Materials and methods

### Human normal and primary tissue samples

Surgically resected tissue samples were obtained under the General Surgery, “Presidio Ospedaliero di Gorizia, Italy approved by the local Ethics Committee. Primary breast tumors were obtained with the appropriate informed consent. Normal breast tissues and corresponding breast tumors were separated at the time of excision, and conserved in RNA Later (Ambion Inc) at – 20°C. The total RNA was extracted using TRIreagent solution (Ambion) followed by an additional cleaning step (RNeasy; Qiagen). RNA was quantified using the Nanodrop spectrophotometer instrument (Thermo Scientific) and quality routinely verified on denaturing agarose gels. Normal breast tissues and tumors were classified according to standard histological evaluations, receptor and proliferation markers as reported in Additional file 3: Table S3.

### Analysis of *TMEM16A* mRNA Splicing isoforms

One μg of total RNA derived from ductal and lobular epithelial breast tumors and normal breast samples and one μg of total RNA derived from 20 human tissues (First Choice Human Total RNA Survey Panel; Ambion) was retro-transcribed in standard conditions and amplified by PCR with a set of primers specific for each alternatively spliced exon: for exon 6b, the sense and antisense primers were 803D^FAM^ and 1385R, 5′-[6FAM]CGGAGCACGATTGTCTATGA and 5’- GGGCCATGAAGACAGAGAAG, respectively, for exon 13, 1368D^FAM^ and 1525R, 5′-[6FAM]TCTCTGTCTTCATGGCCCTC and 5’-CTCCAAGACTCTGGCTTCGT, respectively, and for exon 15, 1506D^FAM^ and 1894R, 5′-[6FAM]ACGAAGCCAGAGTCTTGGAG and 5′-GAACCGATCTCTCCATGTCAGCTTCA, respectively. Conditions for PCR were the following: 94°C for 5 min for the initial denaturation; 94°C for 45 s, 58°C for 45 s, and 72°C for 1 min for 35 cycles; and 72°C for 10 min for the final extension. Semi-quantitative analyses of the *TMEM16A* isoforms were performed by Capillary Electrophoresis (CE) at the BMR genomics, Italy (http://www.bmr-genomics.it/). The relative amount of each mRNA isoform was calculated based on the corrected integrated area of each peak and size was calculated using Rox1000BV marker (Bmr-Genomics). For Spicing Coordination experiments, RNA extracted from human adult normal tissues, normal and tumor breast tissues were amplified using a labelled fluorescent 803D^FAM^ 5′-[6FAM]CGGAGCACGATTGTCTATGA and 1894R primer 5′-GAACCGATCTCTCCATGTCAGCTTCA. Conditions for PCR were the following: 94°C for 3 min for the initial denaturation, 94°C for 45 s, 56°C for 45 s, 72°C for 1 min and 72°C for 10 min for the final extension. 1 μL of the PCR product was dehydrated at the temperature of 60°C for 20 min and sent for the CE analysis. The results are expressed as mean ± S.D. of three independent experiments done in duplicate.

### *TMEM16A* Expression vectors and cell culture

The *TMEM16A* isoforms 6b-13 + 15-, 6b + 13 + 15+ 6b + 13 + 15-, and 6b + 13-15- were obtained form corresponding plasmids [3,22]. *TMEM16A* (6b-13 + 15+) was generated from the *TMEM16A* (6b + 13 + 15+, and 6b-13 + 15-) constructs by RT-PCR (forward) 5′-TGCGACAAGACCTGCAGCTACTGG-3′ and (reverse) 5′-TGTAGGAATTCAAACTTCAGCAG-3′ and cloned in the PstI and EcoRI of pBS(6b-13 + 15-) to generate pBS(6b-13 + 15+). Subsequently, pBS(6b-13 + 15+) was digested with EcoRI and BamHI and cloned in the corresponding sites of pcDNA3.1 to generate pcDNA3.1(6b-13 + 15+). All the coding sequences for *TMEM16A* (6b + 13-15-, 6b + 13 + 15-, 6b + 13 + 15+, 6b-13 + 15-, and 6b-13 + 15+) were cloned in the pcDNA5 FRT/TO plasmid (Invitrogen). Stable expression of *TMEM16A* variants was achieved by Flp-recombinase-mediated recombination in HEK293 Flp-In cells (Invitrogen) followed by hygromycin B selection (InvivoGen). Each *TMEM16A*-expressing vector that expresses the Flp-recombinase (Invitrogen) was co-transfected with Effectene transfection reagent (Qiagen) and selected with a concentration of 200 μ g/ml hygromycin B. Individual clones were obtained by limited dilution. Induction of *TMEM16A* isoforms expression was achieved with 0.1 μg/ml tetracycline (Sigma). Cells were grown in DMEM-Glutamax-I media (GIBCO) supplemented with 5% fetal bovine serum (EuroClone).

### *In vitro* wound healing assay

Cell mobility was assessed using a scratch wound healing assay. HEK293 stable cells were plated (5 × 10^5^ cells) in 6-well plates, coated with poly-D-lysine (1 mg/ml, Sigma). 0.1 μg tetracycline (Sigma) was added one day after plating. Confluent monolayers (24 h after tetracycline induction) were scraped with 200 μl disposable plastic pipette tips. Pictures were taken every 24 h with a confocal microscope (Leica TCS-SL) (4 × amplification). The distances between the wound edges were measured using GraphicConverter software. These experiments were carried out in triplicates.

### Immunofluorescence

*TMEM16A*-inducible HEK293 cells were grown on 6-well plate and cultured in the presence or absence of 0.1 μg/ml tetracycline for 72 hrs to induce *TMEM16A* isoform expression. The cells were fixed with Bouin solution (Sigma-Aldrich) for 10 minutes at room temperature, washed twice in PBS, and incubated with blocking solution (1% BSA and 1% PBS) for 2 hrs at room temperature. The cells were then stained overnight at 4°C with primary monoclonal antibody against *TMEM16A* (Abcam) diluted in 0.3% Triton X-100 (Sigma-Aldrich) in 1%PBS-BSA. Cells were washed with PBS and incubated for 2 h with the respective secondary antibodies conjugated to Alexa Fluor-488 (Life Technologies). This was rinsed again in PBS, before viewing cells under a laser-scanning microscope LSM 5 EXCITER (Carl Zeiss MicroImaging).

### HS-YFP assay

HEK-293 cells, plated in 96-well microplates, were transiently transfected with a plasmid coding for the halidse-sensitive yellow fluorescent protein (HS-YFP) as previously described [3]. After 24 hours, cells were treated with tetracycline to induce *TMEM16A* expression. At the time of assay, cells were washed two times with 100 μl PBS (137 mM NaCl, 2.7 mM KCl, 8.1 mM Na_2_HPO_4_, 1.5 mM KH_2_PO_4_, 1 mM CaCl_2_, and 0.5 mM MgCl_2_; pH = 7.4) and incubated for 30 min with 60 μl PBS. Cells were transferred to the stage of a fluorescence microscope equipped with excitation and emission filters for the yellow fluorescent protein. Cell fluorescence in each well was continuously acquired with a CoolSNAP HQ2 camera before and during addition of 165 μl of modified PBS (Cl^-^ replaced by l^-^; final l- concentration in the well: 100 mM) also containing 1 μM ionomycin. Fluorescence cell traces were normalized to the initial, background-subtracted, fluorescence. *TMEM16A* activity was calculated from the maximal rate of fluorescence quenching caused by l^-^ influx.

### Cell proliferation assay

*TMEM16A*-inducible HEK293 cells, were grown on 96-well plate and cultured in the presence or absence of 0.1 μg/ml tetracycline. After 24 hours, cells were incubated with EdU (Invitrogen). 30 minutes after EdU addition, cells were washed once in PBS, fixed in PFA 4%, for 15 min, permeabilized with 0.5% Triton X-100 in PBS solution for 10 min. EdU detection was carried out using Click-iT EdU Cell Proliferation Assay (Invitrogen).

### Statistical analysis

A statistical analysis was performed on the investigated groups of data using the Student’s t-test and the Fisher’s exact test.

## Abbreviations

AS: Alternative splicing; SC: Splicing coordination.

## Competing interests

The authors declare that they have no competing interests.

## Authors’ contributions

IU and FP conceived the idea and designed the experiments; IU, EB, PS performed the experiments; AC, GS and ML provide human breast and breast cancer tissues and performed histological analysis; IU, LG and FP wrote the manuscript. All authors read and approved the final manuscript.

## Supplementary Material

Additional file 1: Figure S1 Exon-Exon junctions of TMEM16A isoforms. Nucleotide sequences of TMEM16A cDNA isoforms expressed in human adult normal tissues, breast and tumor breast tissues. Click here for file

Additional file 2: Figure S2 Correlation between the percentage of exon 6b and 15 inclusion calculated in short and long RT-PCR amplifications in normal adult human tissues. *A*, schematic representation of the *TMEM16A* mRNA showing the position of the AS exons (black boxes), and the oligonucleotide used in RT-PCR experiments. RNA was amplified with 803DFAM and 1894R; 803DFAM and 1385R; and 1506DFAM and 1894R primers. *B*, (Top) Correlation between the percentage of the long and the short RT-PCR amplification of *TMEM16A* exon 6b. (Bottom) Correlation between the percentage of the long and the short RT-PCR amplification of *TMEM16A* exon 15. Linear regression lines were fitted to the data points obtained from the two amplification systems. Experimental conditions were as described under Materials and Methods. Click here for file

Additional file 3: Table S3 Clinicopathological variables in normal and human breast cancer samples. 36 breast samples obtained by surgical excision where divided in their normal (N) and tumor (T) portions. Samples were classified in virtue of their histological evaluation (second column), D defined as ductal breast or L defined as lobular breast samples; grading (third column), necrosis (forth column) and receptors (fifth, sixth, seventh and eighth column). ER, estrogen receptor; PgR, progesterone receptor; Ki67, nuclear proliferation marker and HER2, human epidermal growth factor receptor 2. The histological evaluation showed 14 ductal and 3 lobular tumors and 1 ductal-bifasic. The majority of tumors are positive for the estrogen and progesterone receptor markers (15 and 13 tissues, respectively). The nuclear proliferation marker Kit67 was low (+1 grading) in most tumors (n = 12) and high (grading +2 and +3) in 6 tumors. The HER2 marker was detected in 5 breast cancers. Click here for file

Additional file 4: Figure S4 TMEM16A isoforms expression in stably transfected HEK293 Flp- In cells. The inducible cell lines were either untreated (left panel) or tetracycline treated (right panel) prior to fluorescence measurement and examined by indirect immunofluorescence analysis using a monoclonal antiserum against *TMEM16A*. Original magnification, 4x. (Scale bars: 5 μm). Click here for file

Additional file 5: Figure S5 Analysis of *TMEM16A* function in HEK293 cells. *A*, representative cell fluorescence traces showing quenching caused by Ca2 + −dependent I- influx. The arrow shows the time of addition of the solution containing high I—and the Ca2+ ionophore ionomycin. *B*, summary of data obtained from multiple experiments. Each bar represents the anion transport (*TMEM16A*) activity expressed as quenching rate (QR) and calculated from the maximal slope of fluorescence decay. Click here for file

Additional file 6: Figure S6 Splicing Coordination of *TMEM16A* mRNA isoforms in normal adult human tissues. *A*, example of a positive (cervix) and a negative (adipose tissues) case of SC. The top of the panel shows the percentage of exon 6b inclusion in transcripts that include or exclude exon 15. Lane 1: percentage of exon 15 inclusion considering all the four isoforms. Lane 2: percentage of exon 15 inclusion in the isoforms that contain only exon 6b. Lane 3: percentage of exon 15 inclusion in the isoforms that lacks exon 6b. The identity of the isoforms considered in each calculation is depicted below each lane. *B*, Percentage of exon 15 inclusion in transcripts that contain or lack exon 6b. Quantification of exon 15 inclusion associated to exon 6b inclusion or exclusion in 20 normal adult human tissues. Statistically analysis was performed using paired Student’s t-test. Ad = Adipose; Bl = Bladder; Cer = Cervix; Col = Colon; Eso = Esophagus; He = Heart; Kid = Kidney; Liv = Liver; Ov = Ovary; Pla = Placenta; Pro = Prostate; Int = Small Intestine; Spl = Spleen; Tes = Testes; Thym = Thymus; Thyr = Thyroid; Tra = Trachea. Click here for file

Additional file 7: Figure S7 Alternative Splicing Coordination of *TMEM16A* in normal and breast cancer tissues. Quantification of exon 6b inclusion associated to exon 15 inclusion or exclusion in normal and breast cancer tissues. The former 18 samples are normal breast tissues, the latter are the corresponding breast cancer tissues. The percentage is expressed as means ± SD, based on at least three independent capillary electrophoresis analyses. Click here for file
