# Supplementary figures and images for: TMEM16A alternative splicing coordination in breast cancer
Source: Mol Cancer. 2013 Jul 16;12:75. doi: 10.1186/1476-4598-12-75 (PMC3728142; doi:10.1186/1476-4598-12-75)

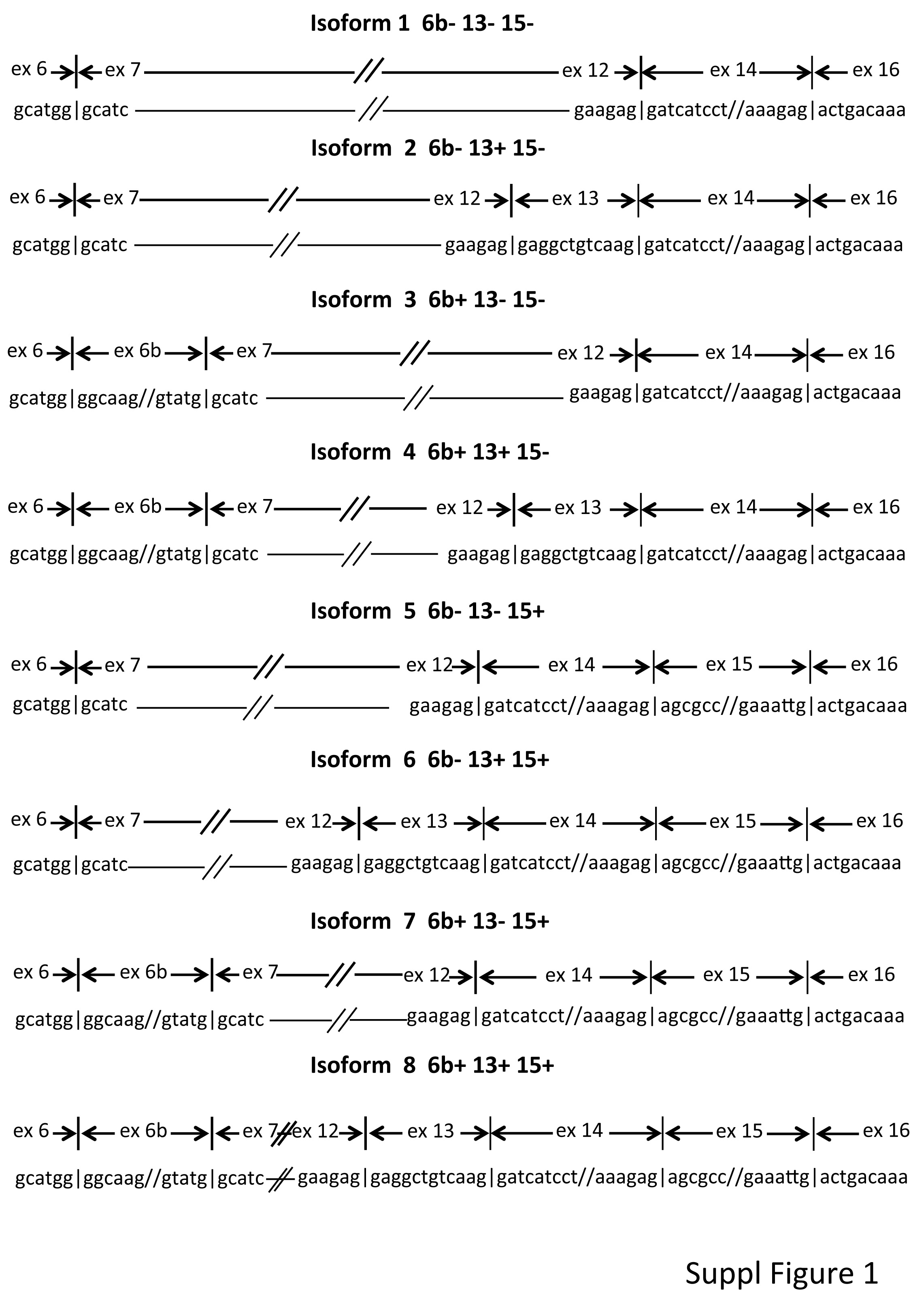

Supplement: Additional file 1: Figure S1 — Exon-Exon junctions of TMEM16A isoforms. Nucleotide sequences of TMEM16A cDNA isoforms expressed in human adult normal tissues, breast and tumor breast tissues. [file 1476-4598-12-75-S1.jpeg]

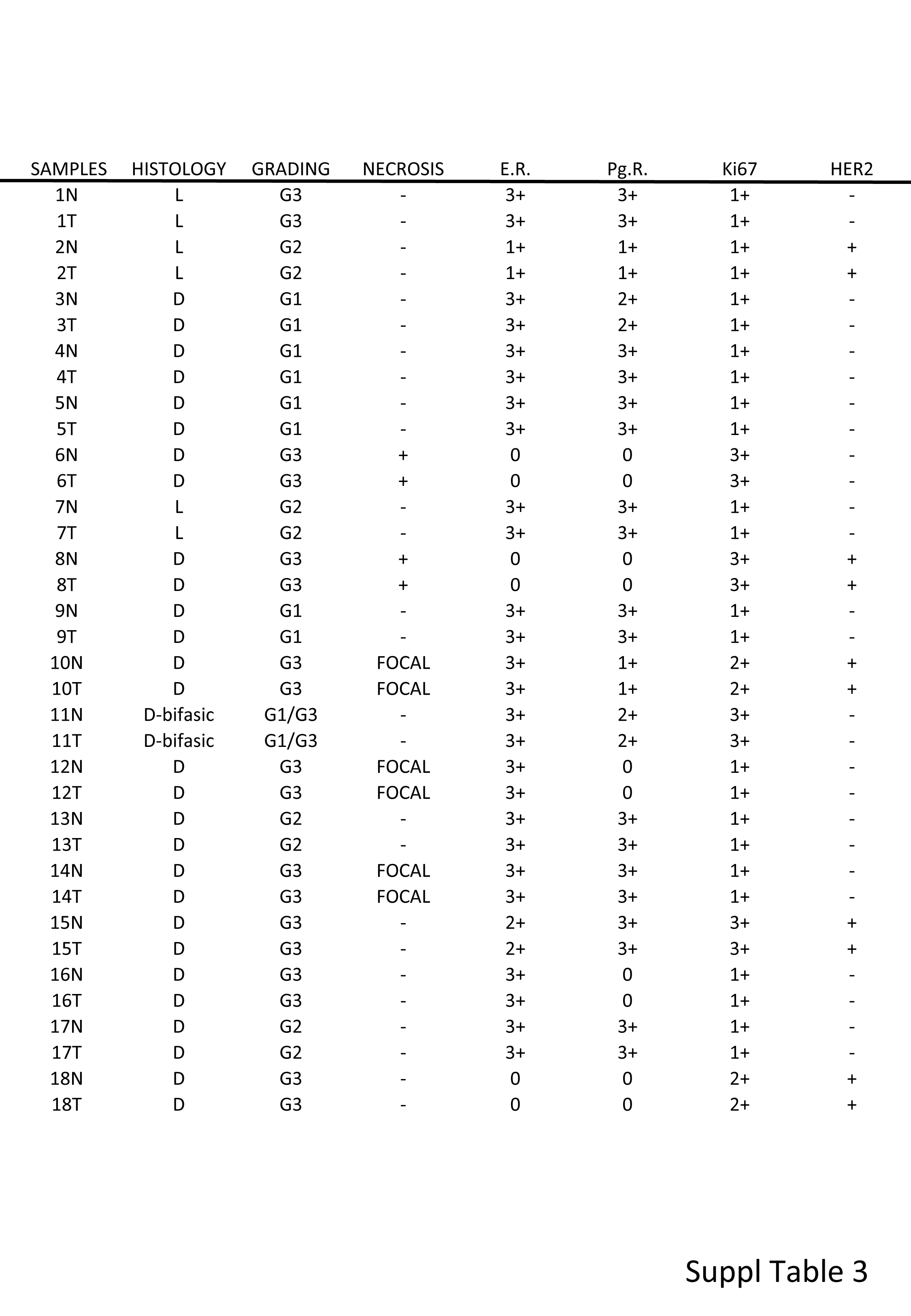

Supplement: Additional file 2: Figure S2 — Correlation between the percentage of exon 6b and 15 inclusion calculated in short and long RT-PCR amplifications in normal adult human tissues. A, schematic representation of the TMEM16A mRNA showing the position of the AS exons (black boxes), and the oligonucleotide used in RT-PCR experiments. RNA was amplified with 803DFAM and 1894R; 803DFAM and 1385R; and 1506DFAM and 1894R primers. B, (Top) Correlation between the percentage of the long and the short RT-PCR amplification of TMEM16A exon 6b. (Bottom) Correlation between the percentage of the long and the short RT-PCR amplification of TMEM16A exon 15. Linear regression lines were fitted to the data points obtained from the two amplification systems. Experimental conditions were as described under Materials and Methods. [file 1476-4598-12-75-S2.jpeg]

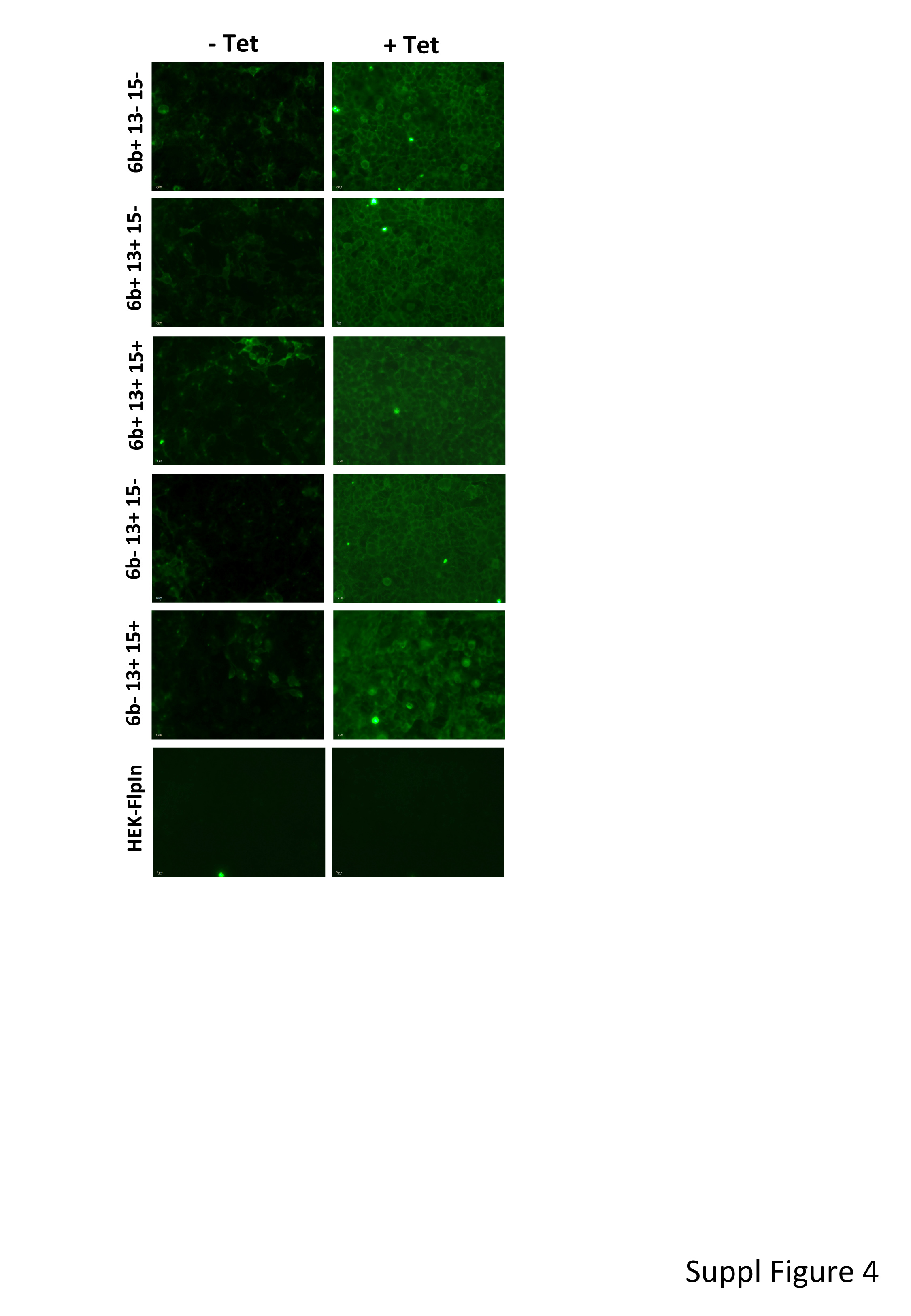

Supplement: Additional file 3: Table S3 — Clinicopathological variables in normal and human breast cancer samples. 36 breast samples obtained by surgical excision where divided in their normal (N) and tumor (T) portions. Samples were classified in virtue of their histological evaluation (second column), D defined as ductal breast or L defined as lobular breast samples; grading (third column), necrosis (forth column) and receptors (fifth, sixth, seventh and eighth column). ER, estrogen receptor; PgR, progesterone receptor; Ki67, nuclear proliferation marker and HER2, human epidermal growth factor receptor 2. The histological evaluation showed 14 ductal and 3 lobular tumors and 1 ductal-bifasic. The majority of tumors are positive for the estrogen and progesterone receptor markers (15 and 13 tissues, respectively). The nuclear proliferation marker Kit67 was low (+1 grading) in most tumors (n = 12) and high (grading +2 and +3) in 6 tumors. The HER2 marker was detected in 5 breast cancers. [file 1476-4598-12-75-S3.jpeg]

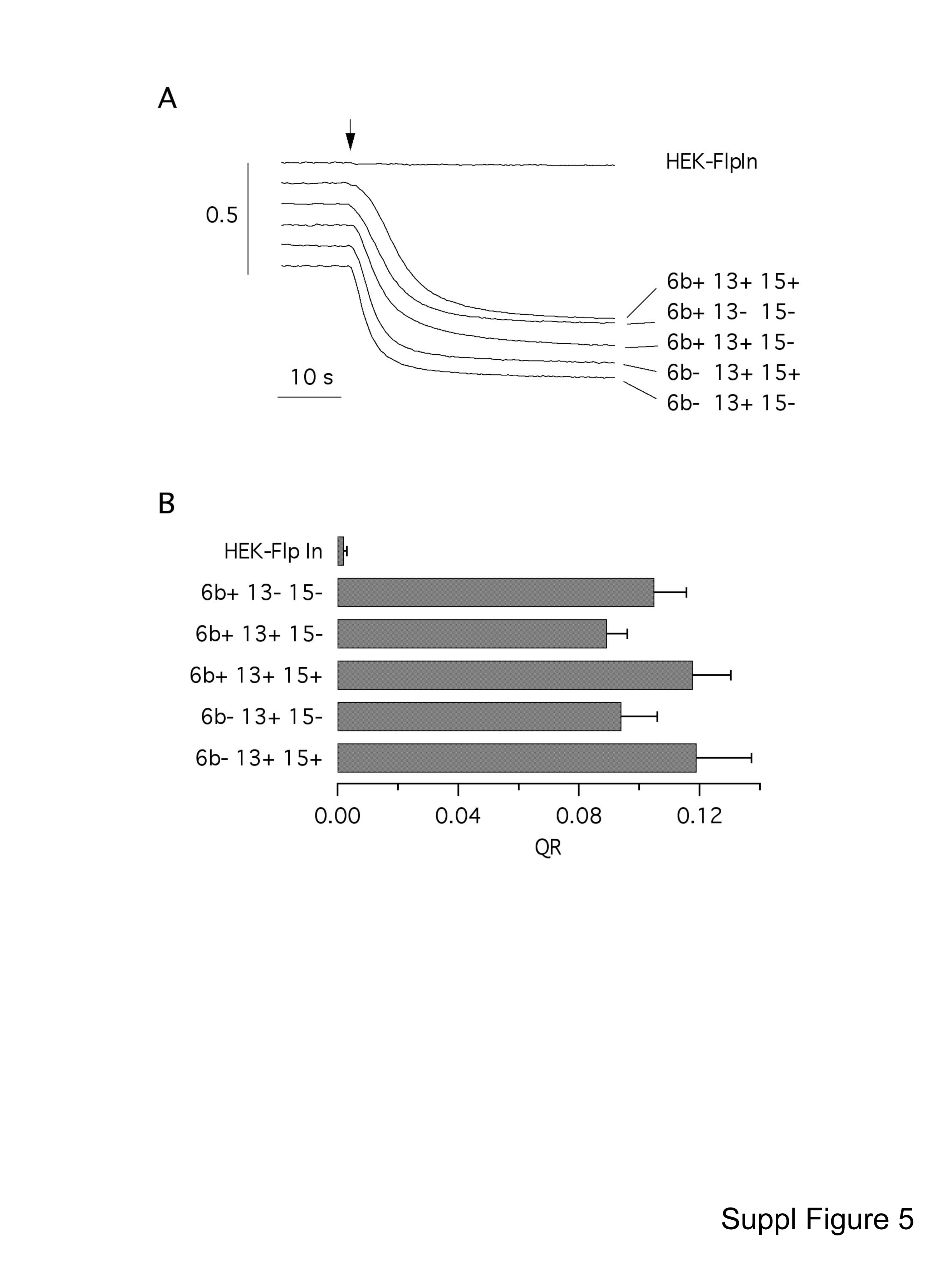

Supplement: Additional file 4: Figure S4 — TMEM16A isoforms expression in stably transfected HEK293 Flp- In cells. The inducible cell lines were either untreated (left panel) or tetracycline treated (right panel) prior to fluorescence measurement and examined by indirect immunofluorescence analysis using a monoclonal antiserum against TMEM16A. Original magnification, 4x. (Scale bars: 5 μm). [file 1476-4598-12-75-S4.jpeg]

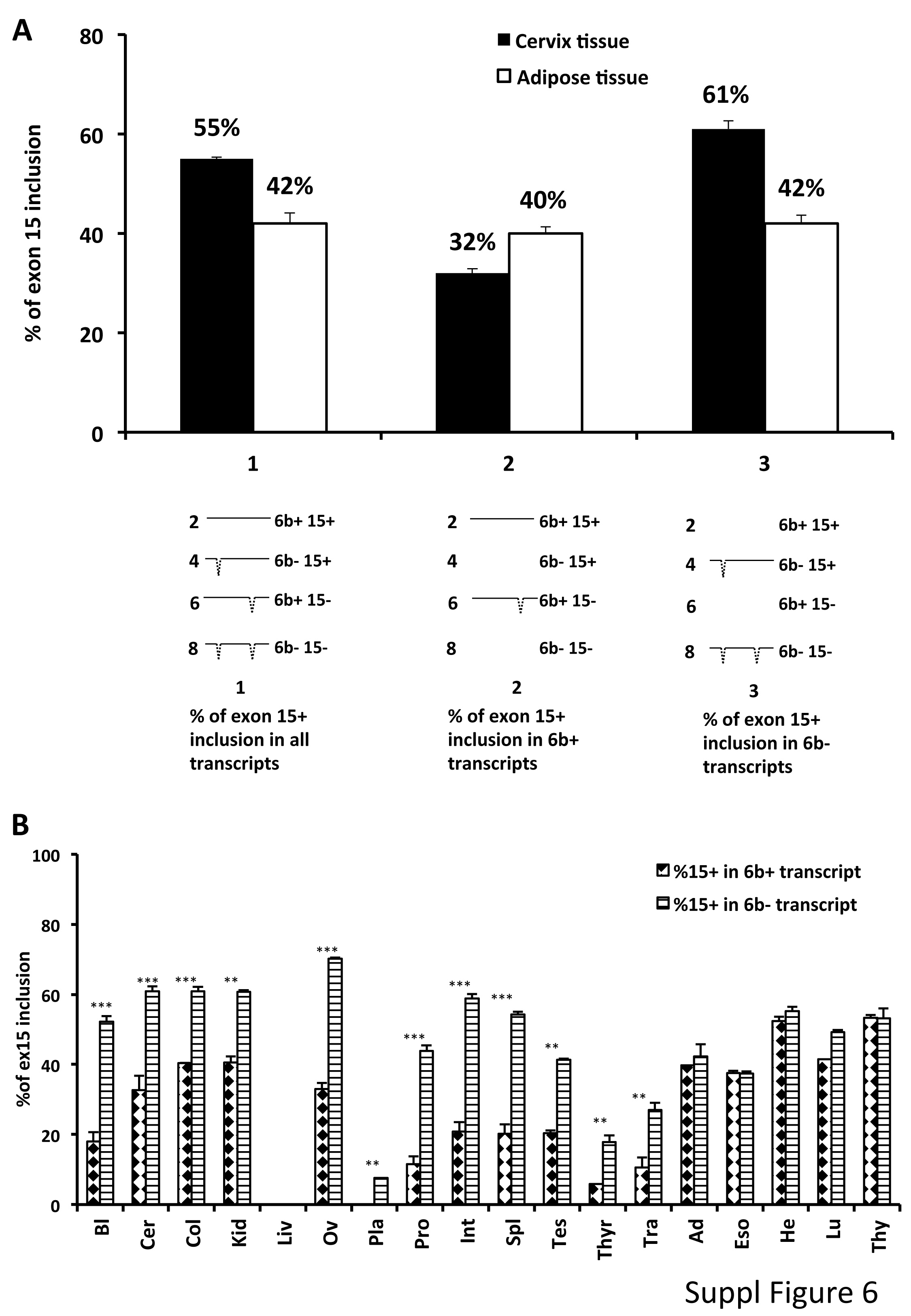

Supplement: Additional file 5: Figure S5 — Analysis of TMEM16A function in HEK293 cells. A, representative cell fluorescence traces showing quenching caused by Ca2 + −dependent I- influx. The arrow shows the time of addition of the solution containing high I—and the Ca2+ ionophore ionomycin. B, summary of data obtained from multiple experiments. Each bar represents the anion transport (TMEM16A) activity expressed as quenching rate (QR) and calculated from the maximal slope of fluorescence decay. [file 1476-4598-12-75-S5.jpeg]

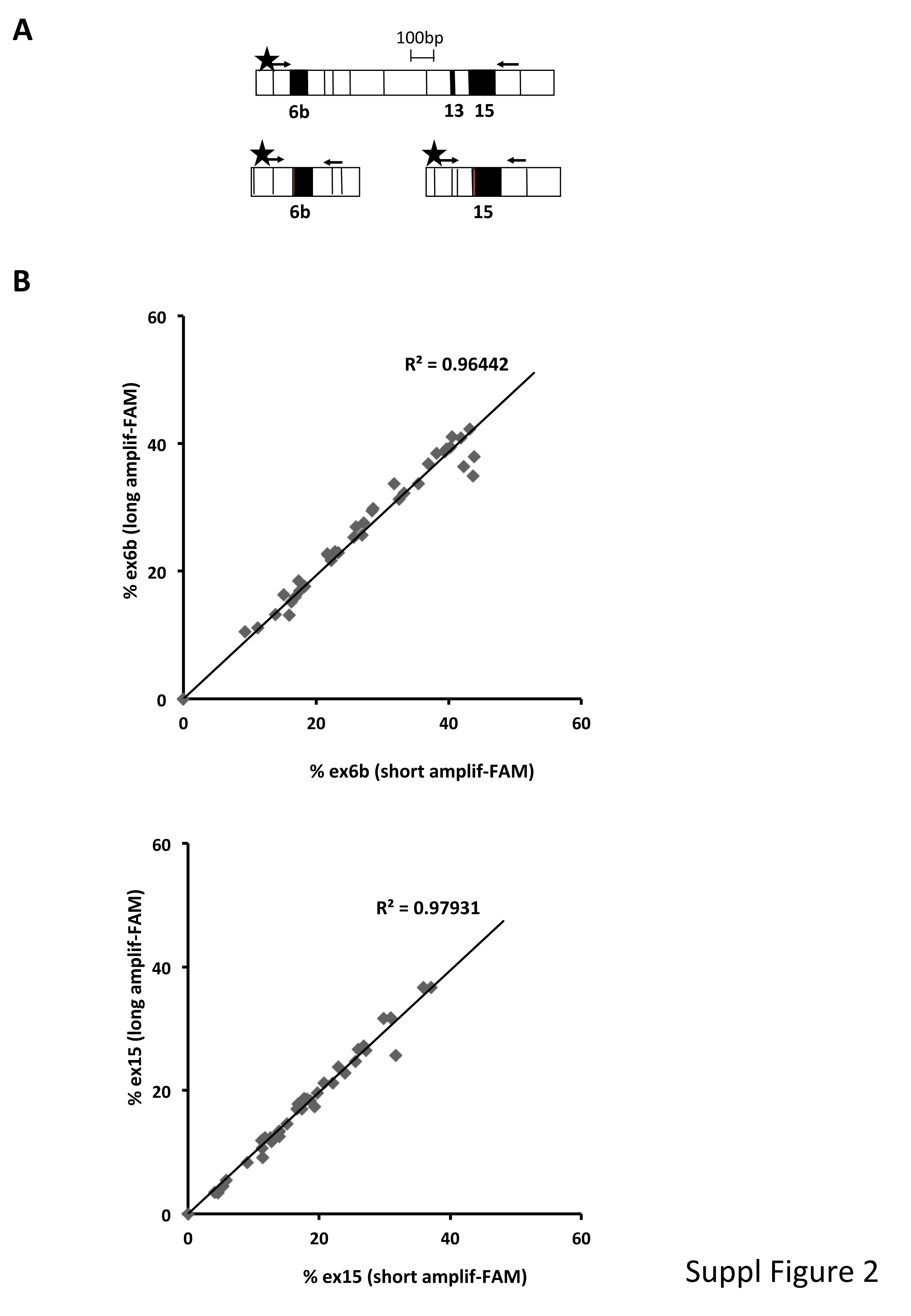

Supplement: Additional file 6: Figure S6 — Splicing Coordination of TMEM16A mRNA isoforms in normal adult human tissues. A, example of a positive (cervix) and a negative (adipose tissues) case of SC. The top of the panel shows the percentage of exon 6b inclusion in transcripts that include or exclude exon 15. Lane 1: percentage of exon 15 inclusion considering all the four isoforms. Lane 2: percentage of exon 15 inclusion in the isoforms that contain only exon 6b. Lane 3: percentage of exon 15 inclusion in the isoforms that lacks exon 6b. The identity of the isoforms considered in each calculation is depicted below each lane. B, Percentage of exon 15 inclusion in transcripts that contain or lack exon 6b. Quantification of exon 15 inclusion associated to exon 6b inclusion or exclusion in 20 normal adult human tissues. Statistically analysis was performed using paired Student’s t-test. Ad = Adipose; Bl = Bladder; Cer = Cervix; Col = Colon; Eso = Esophagus; He = Heart; Kid = Kidney; Liv = Liver; Ov = Ovary; Pla = Placenta; Pro = Prostate; Int = Small Intestine; Spl = Spleen; Tes = Testes; Thym = Thymus; Thyr = Thyroid; Tra = Trachea. [file 1476-4598-12-75-S6.jpeg]

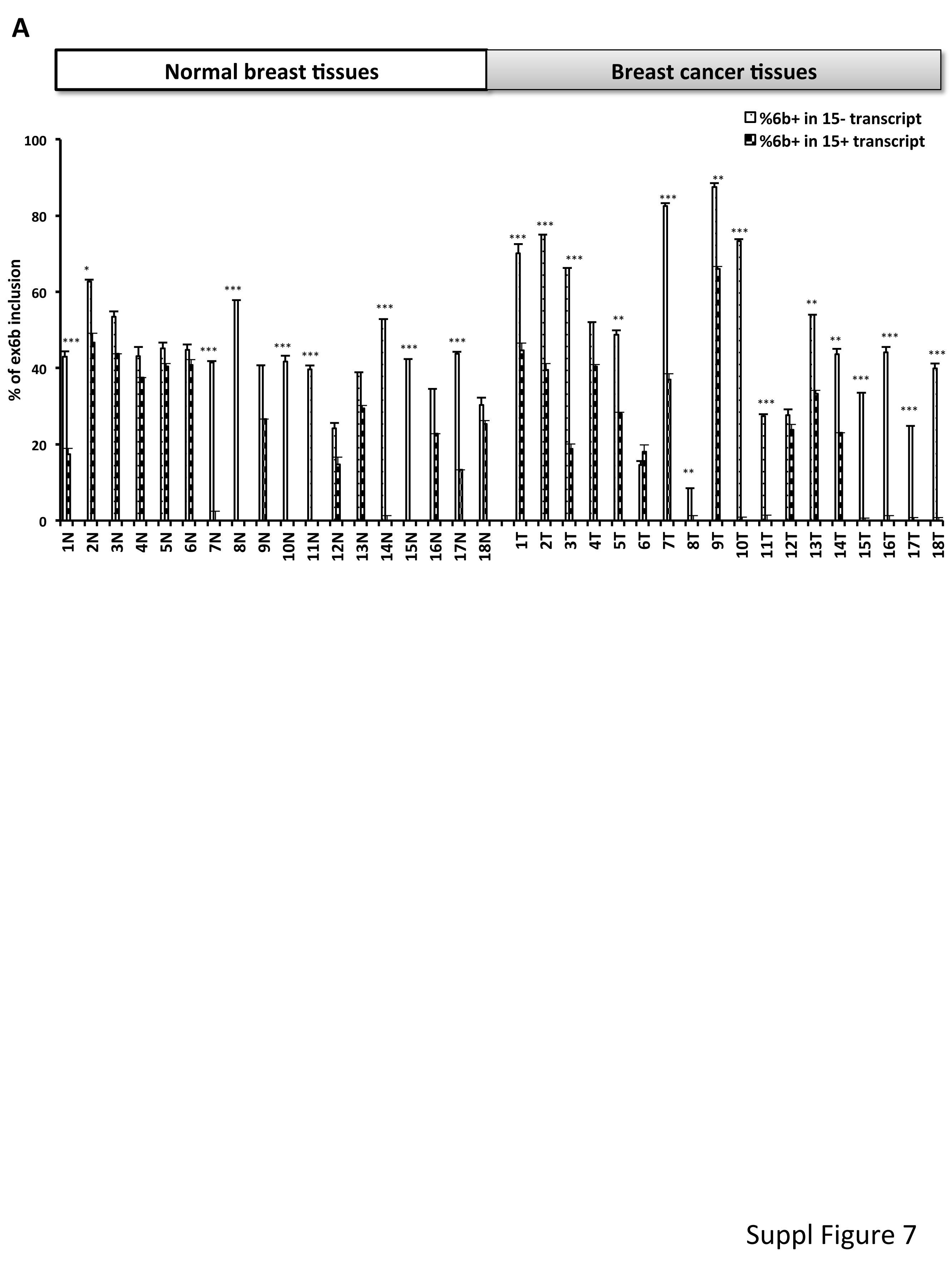

Supplement: Additional file 7: Figure S7 — Alternative Splicing Coordination of TMEM16A in normal and breast cancer tissues. Quantification of exon 6b inclusion associated to exon 15 inclusion or exclusion in normal and breast cancer tissues. The former 18 samples are normal breast tissues, the latter are the corresponding breast cancer tissues. The percentage is expressed as means ± SD, based on at least three independent capillary electrophoresis analyses. [file 1476-4598-12-75-S7.jpeg]
